# Therapeutic Effects and Mechanisms of Sodium New Houttuyfonate in a Murine Model of Intra-Abdominal *Candida albicans* Infection

**DOI:** 10.3390/ijms27146437

**Published:** 2026-07-20

**Authors:** Xiaoyu Peng, Yuxin Xie, Rong Wang, Wenjing Chen, Hong Yu, Zhangyong Song

**Affiliations:** 1 School of Basic Medical Sciences, Southwest Medical University, Luzhou 646000, China; pengxy1216@163.com (X.P.); xieyuxin0612@163.com (Y.X.); wangrong_swmu@163.com (R.W.); 17711368979@163.com (W.C.); 2 School of Nursing, Southwest Medical University, Luzhou 646000, China; 3 Department of Medical Laboratory, Xi’an Guoyi Hospital, Xi’an 712000, China; 4 Public Center of Experimental Technology, Southwest Medical University, Luzhou 646000, China

**Keywords:** sodium new houttuyfonate, *Candida albicans*, intra-abdominal candidiasis, macrophage polarization

## Abstract

Excessive use of immunosuppressive agents compromises host immune defenses and broad-spectrum antimicrobial drugs disrupts the normal microbiota, thereby promoting the overgrowth and dissemination of *Candida albicans*. As an opportunistic pathogen that commonly resides in the intestinal microbiota, *C. albicans* can subsequently translocate across the intestinal barrier and cause intra-abdominal infections. To investigate this process, a murine model of peritoneal *C. albicans* infection was established, in which sodium new houttuyfonate was administered for therapeutic evaluation. The therapeutic potential of sodium new houttuyfonate against abdominal *C. albicans* infection was evaluated through assessment of immune cell composition, peritoneal macrophage polarization, tissue fungal burden, and histopathological features. The molecular mechanisms of sodium new houttuyfonate therapy were also investigated with cellular experiments, including colony counting, real-time quantitative PCR, Western blotting, and the detection of reactive oxygen species (ROS) in RAW264.7 macrophages. Our results revealed that sodium new houttuyfonate exerts a dual anti-infective effect through its fungicidal activity and via the immunomodulation of immunoinflammatory states. Sodium new houttuyfonate also stimulates cytokine production (e.g., IL-1β, IL-6, IL-10, TNF-α, and MCP-1) via the TLR2/p38/NF-κB pathway and promotes the release of ROS and nitric oxide. Overall, these findings highlight the potential of exogenous sodium new houttuyfonate as a therapeutic option for abdominal *C. albicans* infection.

## 1. Introduction

*Candida albicans* is an opportunistic fungal pathogen capable of causing invasive infections. Systemic candidiasis can arise through multiple routes, including disruption of mucosal barriers, colonization of indwelling medical devices, postoperative contamination, and translocation across the gastrointestinal tract. Among these, gastrointestinal translocation is considered a major pathogenic route leading to intra-abdominal candidiasis [1,2,3]. Intra-abdominal *C. albicans* infection is a serious clinical condition with high morbidity and mortality rates, and is especially prevalent among patients undergoing cancer chemotherapy, organ transplantation, or long-term immunosuppressive therapy [4]. In recent years, the widespread use of broad-spectrum antibiotics has made fungal drug resistance a major global public health challenge, particularly in hospital settings, where the incidence of drug-resistant fungal infections has been rising annually and has become a significant problem for clinical treatments [5]. Specifically, the resistance of common fungi such as *C. albicans* to conventional antifungal agents, such as amphotericin B and fluconazole, has been increasing continuously. This phenomenon significantly impedes the effectiveness of treatment. The rising incidence of drug-resistant *C. albicans* infections poses unprecedented challenges to clinical care, especially for immunosuppressed patients. The broad dissemination of drug-resistant *C. albicans* has recently caused a substantial decline in the efficacy of traditional antifungal drugs, further intensifying the difficulty in treating these infections [6,7]. Therefore, there is an urgent need to develop novel antifungal agents to tackle this problem.

Sodium new houttuyfonate is a chemically synthesized derivative of houttuynin, the principal antimicrobial constituent of *Houttuynia cordata*, a medicinal herb widely used in traditional Chinese medicine. Compared with sodium houttuyfonate, sodium new houttuyfonate exhibits improved chemical stability and possesses a broad spectrum of biological activities, including antibacterial, antifungal, anti-inflammatory, and immunomodulatory effects [8,9]. Chinese patent medicines are an important part of traditional Chinese medicine, which involves abundant natural medicinal resources and a profound theoretical foundation [10]. In recent years, with the robust development of traditional Chinese medicines, research into these medicines has become an academic hotspot [11]. Given their unique advantages, including their natural compositions, low toxicity, immunoregulatory activities, and their ability to avoid the development of drug resistance [5]. Chinese patent medicines have shown significant efficacy in immunomodulation, as well as antifungal and anti-inflammatory properties [12,13]. For example, *Houttuynia cordata* Thunb. has been classified by the National Health Commission of China as a medicinal plant resource with “medicine–food homology” [14,15]. Previous studies have demonstrated that sodium new houttuyfonate significantly reduces the bacterial load in *Pseudomonas aeruginosa* infection models by modulating the toll-like receptor 4 (TLR4)/nuclear factor κB (NF-κB) signaling pathway in mouse peritoneal macrophages [16]. Research has also indicated that sodium new houttuyfonate directly inhibits pathogens such as *Staphylococcus aureus*, *Streptococcus* spp., and fungal species such as *C. albicans*, *Aspergillus fumigatus*, and *A. flavus* [17,18,19,20,21]. In recent studies, we have shown that sodium new houttuyfonate not only exerts strong antibacterial activity but also enhances immune capacity by activating macrophage functions [17,20,22]. However, no research has yet identified the specific pathways through which sodium new houttuyfonate synergistically enhances antifungal effects via immunomodulatory mechanisms, and particularly its role in regulating the macrophage–neutrophil immune axis.

Macrophages are essential to immune processes, especially in antifungal responses [23]. As key components of the innate immune response, they not only engulf and eliminate pathogens but also secrete chemokines to recruit neutrophils, thereby enhancing fungicidal efficacy [24]. Macrophages recognize fungal pathogens by detecting pathogen-associated molecular patterns (PAMPs), such as cell wall β-glucan, through pattern recognition receptors (PRRs) on their surface, including Toll-like receptors (TLRs) [25]. This recognition activates downstream signaling pathways, such as mitogen-activated protein kinase (MAPK) and nuclear factor kappa-B (NF-κB) pathways, leading to cytokine secretion and the recruitment of other immune cells, which exert synergistic antifungal effects [26]. However, macrophage activity can be suppressed in immunosuppressed individuals, causing immune tolerance and failure to effectively eradicate pathogens [22,27]. Therefore, restoring and enhancing macrophage activity is critical for the defense against *C. albicans* infections. Several studies have shown that pretreatment with sodium houttuyfonate significantly enhances macrophage phagocytic activity and improves the host’s immune defenses against fungal infections [28,29]. Sodium houttuyfonate also exerts immunomodulatory effects by suppressing an excessive inflammatory response and alleviating infection-induced tissue damage [30]. In this work, we explored the effects of sodium new houttuyfonate in a model of intraperitoneal *C. albicans* infection, paying particular attention to how it influences macrophage activity and host immune responses. Our findings provide a new theoretical foundation for the treatment of drug-resistant fungal infections and innovative approaches to the clinical application of herbal medicines to intra-abdominal *C. albicans* infections.

## 2. Results

### 2.1. In Vivo Therapeutic Efficacy of Sodium New Houttuyfonate in the Intra-Abdominal Candidiasis Model

To evaluate the therapeutic potential of sodium new houttuyfonate against intra-abdominal *C. albicans* infection, a murine infection model was established to assess survival outcomes, fungal burden, and pathological changes following sodium new houttuyfonate treatment. Following 3 days of treatment, pathological changes in the liver and kidney were notably improved in both the sodium new houttuyfonate and fluconazole (FCZ) groups compared with the untreated *C. albicans*-infected (Ca) group, as reflected by decreased inflammatory infiltration and less pronounced mesangial expansion, although slight abnormalities remained. After treatment for 7 days, inflammatory infiltration was alleviated, with improvement in the infection-induced tissue damage. Prominent lymphoid follicle structures were also observed in the small-intestinal tissues of the Ca group, whereas no such structures were detected in the sodium new houttuyfonate or FCZ treatment group. Further examination of the small-intestinal tissues revealed that the sodium new houttuyfonate and FCZ groups displayed only mild villous damage, little inflammatory cell infiltration, and a relatively intact tissue architecture.

**Figure 1:**
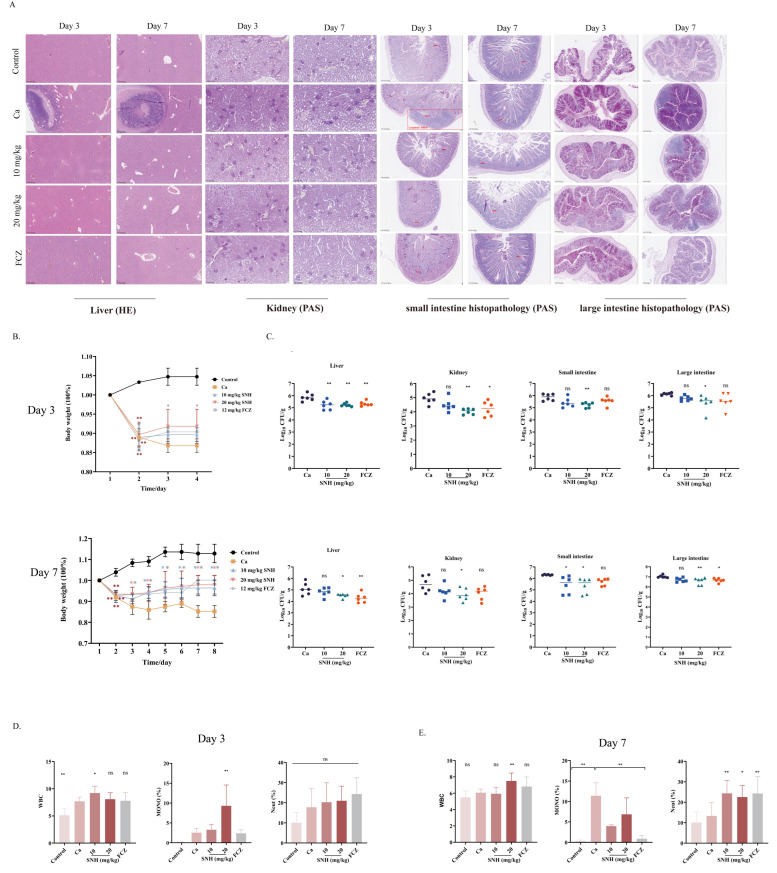
*In vivo* therapeutic efficacy of sodium new houttuyfonate in a murine model of invasive candidiasis. (**A**) Fungal burdens in major organ tissues were assessed with Periodic acid–Schiff staining (scale bar: 50 μm). (**B**) Bodyweight changes in mice of different treatment groups (n = 6) during the infection period. (**C**) Quantitative analysis of fungal loads in various organs on days 3 and 7 post-treatment. Flow-cytometric analysis of the percentage changes in peripheral white blood cells (WBC), monocytes, and neutrophils on day 3 (**D**) and day 7 (**E**) of treatment. FCZ indicates fluconazole treatment. Data were analyzed with one-way ANOVA. ns: not significant; * *p* < 0.05, ** *p* < 0.01, *** *p* < 0.001, significantly different from *C. albicans*-infected group (Ca).

Periodic acid–Schiff (PAS) staining showed that fungal structures stained in magenta were less frequently detected after sodium new houttuyfonate treatment than in the Ca group, while the control group exhibited a relatively higher presence of these fungal colonies (Figure 1A). Moreover, all *C. albicans*-infected mice showed significantly lower body weights than the control mice. Compared with the Ca group, mice receiving sodium new houttuyfonate or FCZ showed steady weight gain over 3 and 7 days of treatment (Figure 1B). The fungal burden in the liver was also significantly lower after treatment with 10 mg/kg or 20 mg/kg sodium new houttuyfonate for 3 days. Furthermore, the renal and small-intestinal fungal burdens were markedly reduced in the 20 mg/kg sodium new houttuyfonate group. After sodium new houttuyfonate treatment for 7 days, the fungal burdens in the liver, kidneys, small intestine, and large intestine were significantly reduced in the 20 mg/kg sodium new houttuyfonate group relative to the control, whereas the small-intestinal fungal burden was significantly reduced in the 10 mg/kg houttuyfonate-treated group (Figure 1C).

To explore the contribution of the immune system to sodium new houttuyfonate treatment, blood profiles were assessed. After 3 days of administration, mice receiving 10 or 20 mg/kg sodium new houttuyfonate showed higher proportions of leukocytes and monocytes than those in the Ca group. The percentages of neutrophils also showed upward trends in the 10 mg/kg and 20 mg/kg sodium new houttuyfonate-treated groups (Figure 1D). After continuous treatment for 7 days, the white blood cell counts were significantly elevated in the 20 mg/kg sodium new houttuyfonate-treated group compared with the Ca group, but the percentage of monocytes was significantly lower than in the Ca group (Figure 1E). In contrast, the neutrophil counts were significantly higher than in the Ca group (Figure 1D,E).

### 2.2. Sodium New Houttuyfonate Enhances Macrophage Antifungal Activity Without Direct Fungicidal Effects

**Figure 2:**
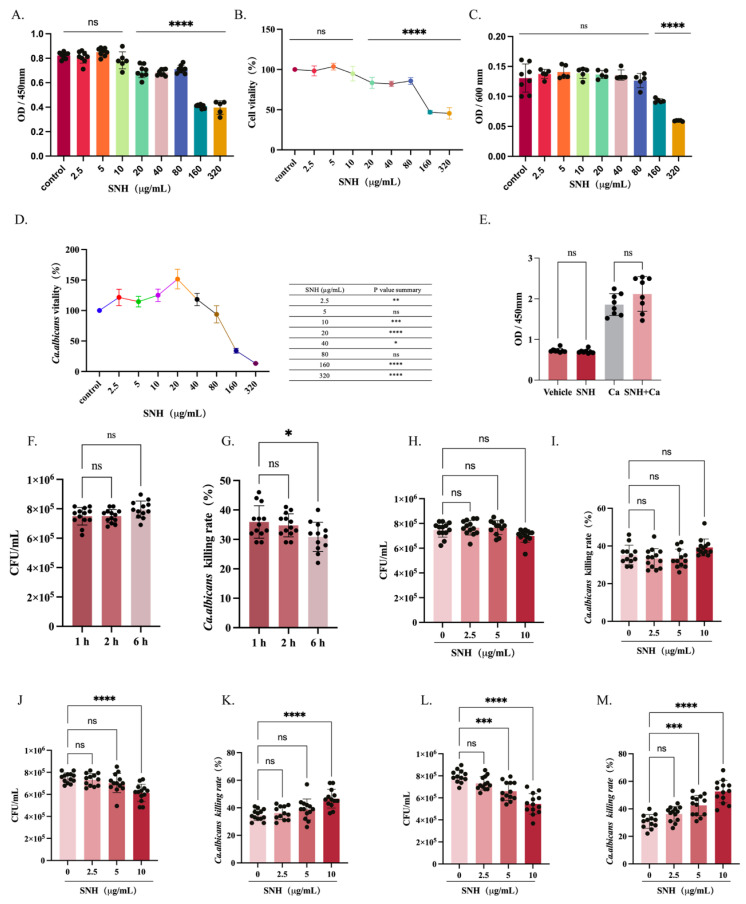
Sodium new houttuyfonate (SNH) exerts antifungal effects by enhancing macrophage function rather than through direct fungicidal activity. (**A**) OD_450_ and (**B**) viability of RAW264.7 cells after 6 h in culture with sodium new houttuyfonate (≤80 μg/mL), as determined with a CCK-8 assay. Macrophage viability exceeded 95% at sodium new houttuyfonate concentrations below 10 μg/mL, establishing 2.5–10 μg/mL as the safe concentration range. (**C**) Optical density (OD_600_) and (**D**) fungal growth inhibition rate of *C. albicans* after 6 h in culture with sodium new houttuyfonate at various concentrations (≤80 μg/mL). (**E**) XTT assay evaluating the effect of 10 μg/mL sodium new houttuyfonate on fungal metabolic activity. Vehicle: YPD medium containing 0.05% Tween-80; sodium new houttuyfonate: Vehicle supplemented with 10 μg/mL sodium new houttuyfonate; Ca: Vehicle supplemented with *C. albicans* (1 × 10^6^ cells/mL); sodium new houttuyfonate + Ca: Vehicle supplemented with both sodium new houttuyfonate and *C. albicans*. One-way ANOVA showed no significant differences compared with the untreated group. (**F**,**G**) Fungal clearance efficiency in RAW264.7 cells gradually decreased over time after co-culture with *C. albicans*. (**H**,**I**) No significant differences were observed between the sodium new houttuyfonate-treated groups and the Ca group (0 μg/mL) after sodium new houttuyfonate intervention for 1 h. (**J**,**K**) After treatment for 2 h, 10 μg/mL sodium new houttuyfonate increased fungal killing efficiency 2.3-fold relative to that in the Ca group (0 μg/mL). (**L**,**M**) After treatment for 6 h, 5 μg/mL and 10 μg/mL sodium new houttuyfonate enhanced the measured parameters 1.8-fold and 2.5-fold, respectively, relative to the Ca group (0 μg/mL). Data were analyzed with one-way ANOVA. ns: not significant; * *p* < 0.05, ** *p* < 0.01, *** *p* < 0.001, **** *p* < 0.0001, significantly different from Ca group.

To determine whether the protective effects of sodium new houttuyfonate against *C. albicans* infection are attributable to direct antifungal activity or host immune modulation, the direct inhibitory effects of sodium new houttuyfonate on fungal growth were first evaluated *in vitro*. Before investigating the immunomodulatory effects of sodium new houttuyfonate on macrophage-mediated antifungal responses, its cytotoxicity toward RAW264.7 macrophages was assessed using a Cell Counting Kit-8 (CCK-8) assay. Cell viability remained above 95% after treatment with sodium new houttuyfonate at concentrations below 10 μg/mL, indicating negligible cytotoxicity within this concentration range. Therefore, sodium new houttuyfonate concentrations of 2.5–10 μg/mL were selected for subsequent functional experiments (Figure 2A,B).

To assess whether sodium new houttuyfonate exerts direct antifungal activity under the conditions used for macrophage co-culture experiments, a short-term *C. albicans* proliferation assay was performed. Treatment with sodium new houttuyfonate at concentrations up to 80 μg/mL for 6 h did not significantly affect fungal growth compared with the untreated control group (one-way ANOVA, *p* > 0.05). These findings indicate that sodium new houttuyfonate does not exhibit detectable direct growth-inhibitory activity against *C. albicans* during the experimental period, suggesting that its protective effects are likely mediated primarily through host immune regulation rather than direct fungal killing (Figure 2C,D).

Because 10 μg/mL was the highest non-cytotoxic concentration selected for macrophage functional assays, an additional XTT reduction assay was performed to further evaluate its potential direct effects on fungal viability. No significant difference in metabolic activity was observed between the Vehicle and sodium new houttuyfonate groups. Similarly, the metabolic activity of *C. albicans* did not differ significantly between the Ca and sodium new houttuyfonate + Ca groups after 6 h of incubation (Figure 2E). These findings indicate that 10 μg/mL sodium new houttuyfonate does not directly impair the viability or metabolic activity of *C. albicans* under the conditions used for macrophage co-culture.

Taken together, these results suggest that the effects of sodium new houttuyfonate observed in subsequent experiments are unlikely to result from direct antifungal activity and are more likely attributable to modulation of macrophage function.

### 2.3. Quantitative Colony-Forming Units (CFUs) Analysis Confirms Enhanced Macrophage Phagocytic Activity

To explore whether sodium new houttuyfonate enhances macrophage-mediated antifungal defense, macrophage phagocytosis and intracellular killing activity against *C. albicans* were assessed following sodium new houttuyfonate treatment. Following the co-culture of RAW264.7 cells with *C. albicans*, the efficiency of fungal clearance decreased progressively over time. The phagocytosis-mediated killing rates declined from 35% at 1 h to 31% at 6 h, suggesting potential time-dependent exhaustion of the macrophage antimicrobial function (Figure 2F,G).

To evaluate the enhancing effect of sodium new houttuyfonate on the fungal clearance capacity of macrophages, we treated *C. albicans*-engulfed macrophages with varying concentrations (0–10 μg/mL) of sodium new houttuyfonate. At 1 h after treatment, the sodium new houttuyfonate-treated groups showed no clear differences compared with the Ca group. (Figure 2H,I). After treatment for 2 h, 10 μg/mL sodium new houttuyfonate increased the fungal killing efficiency 2.3-fold relative to that of the control group (*p* < 0.01) (Figure 2J,K). After treatment for 6 h, the killing efficiency in the 5 μg/mL and 10 μg/mL sodium new houttuyfonate groups was significantly enhanced, 1.8-fold and 2.5-fold, respectively, relative to that of the control (both *p* < 0.001) (Figure 2L,M). In conclusion, these findings indicate that sodium new houttuyfonate significantly enhances the antifungal function of macrophages.

### 2.4. Sodium New Houttuyfonate Enhances Macrophage Antifungal Function Through the Temporal Regulation of Reactive Oxygen Species (ROS), Nitric Oxide (NO), and Cytokine Expression

**Figure 3:**
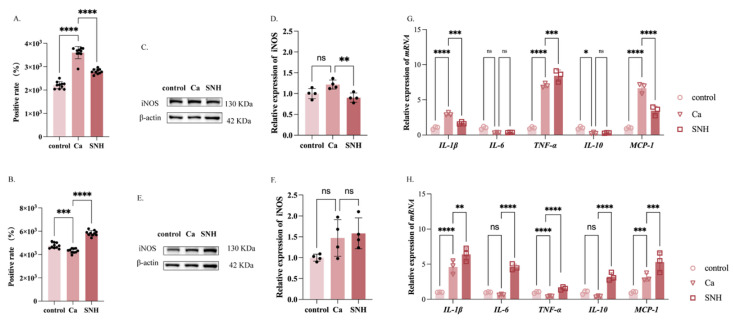
Sodium new houttuyfonate (SNH) enhances macrophage antifungal function through the temporal regulation of ROS, NO, and cytokine expression. (**A**) DCFH-DA probe analysis showed that *C. albicans* infection for 2 h elicited a ROS burst in macrophages, whereas the sodium new houttuyfonate intervention rapidly reduced the fluorescence intensity. (**B**) At 6 h after infection, the reduced fluorescence intensity in the Ca group suggested the development of immune tolerance, whereas the sodium new houttuyfonate treatment group showed a rapid dose-dependent increase in fluorescence intensity. (**C**) iNOS protein expression and (**D**) grayscale analysis after sodium new houttuyfonate treatment for 2 h. Sodium new houttuyfonate downregulated iNOS expression at 2 h postinfection, demonstrating an early anti-inflammatory role. (**E**) iNOS protein expression and (**F**) grayscale analysis after sodium new houttuyfonate treatment for 6 h. At this later time point, sodium new houttuyfonate treatment promoted iNOS upregulation, which enhanced antifungal function during the immune tolerance phase. (**G**) Expression of related inflammatory cytokine genes after sodium new houttuyfonate treatment for 2 h. (**H**) Expression of related inflammatory cytokines after sodium new houttuyfonate treatment for 6 h. Data were analyzed with one-way ANOVA. ns: not significant; * *p* < 0.05, ** *p* < 0.01, *** *p* < 0.001, **** *p* < 0.0001.

To clarify the molecular mechanisms by which sodium new houttuyfonate modulates the antifungal function of macrophages, we focused in this study on the effects of sodium new houttuyfonate on ROS generation. Using 2’,7’-dichlorodihydrofluorescein diacetate (DCFH-DA) probe labeling, the fluorescence intensity of DCF (excitation/emission: 488/525 nm) was continuously monitored with a fluorescence microplate reader. At 2 h, fluorescence intensity was higher in theCa group than in the control group (*p* < 0.05), suggesting that infection induces a burst of ROS in macrophages. After the sodium new houttuyfonate intervention, the fluorescence intensity declined rapidly, demonstrating that sodium new houttuyfonate mitigates excessive inflammation during the early stage of infection (Figure 3A). By 6 h, the fluorescence intensity in the Ca group had decreased relative to that in the control group, indicating the development of immune tolerance in the macrophages over time. After treatment with sodium new houttuyfonate, the fluorescence intensity increased rapidly. This dose-dependent effect was closely associated with the enhanced antifungal activity observed in previous studies, suggesting a positive association between enhanced ROS production and the increased antifungal activity observed following sodium new houttuyfonate treatment (Figure 3B). Given the critical role of NO in the inhibition of fungal growth and pathogen clearance, we used Western blotting to investigate whether sodium new houttuyfonate influences NO production by examining the expression of inducible nitric oxide synthase (iNOS). The sodium new houttuyfonate treatment downregulated iNOS expression at 2 h, suggesting that sodium new houttuyfonate exerts anti-inflammatory effects during early infection by reducing iNOS expression (Figure 3C,D). In contrast, at 6 h, sodium new houttuyfonate treatment increased iNOS expression, indicating that sodium new houttuyfonate enhances the antifungal function of macrophages during the immune tolerance phase by upregulating iNOS expression (Figure 3E,F). During the early stage of infection (2 h), *C. albicans* stimulation significantly upregulated the levels of proinflammatory cytokines, such as *IL-1β*, *TNF-α*, and *MCP-1*, in macrophages, indicating the rapid initiation of the inflammatory response. After the sodium new houttuyfonate intervention, the expression of IL-1β and MCP-1 decreased, whereas TNF-α expression was further upregulated (Figure 3G). By the late stage of infection (6 h), the sodium new houttuyfonate treatment not only maintained elevated IL-1β levels but also further upregulated the expression of *IL-6*, *IL-10*, *MCP-1*, and *TNF-α* (Figure 3H).

### 2.5. Dual Immunomodulatory Effects of Sodium New Houttuyfonate on Peritoneal Macrophages

**Figure 4:**
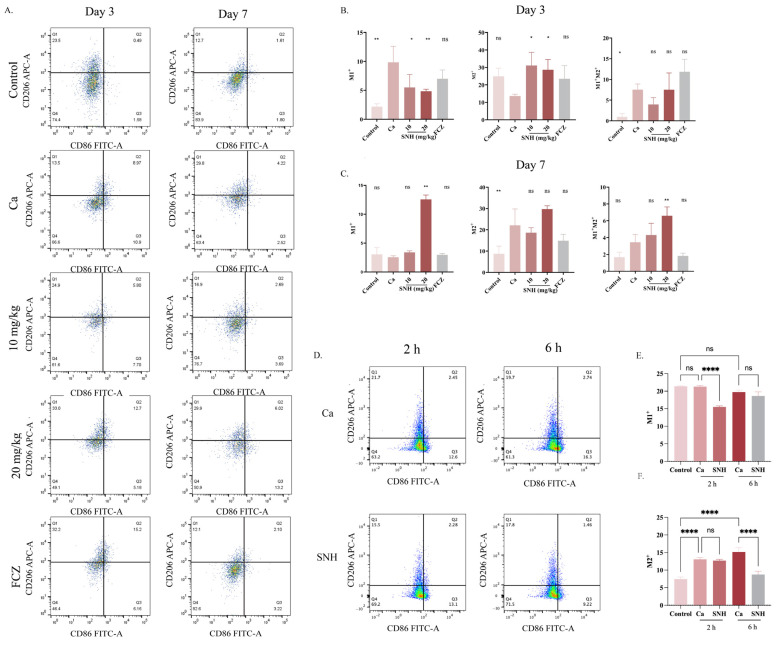
Sodium new houttuyfonate (SNH) exerts dual immunomodulatory effects through the temporal regulation of macrophage phenotypic switching. (**A**) In vivo polarization of peritoneal macrophages. Mice were infected intraperitoneally with *C. albicans* for 2 h and then treated with sodium new houttuyfonate for 3 or 7 days, using FCZ as the positive control. Representative flow-cytometric plots show the polarization changes in peritoneal macrophages. Color scale represents cell density (events). (**B**,**C**) Statistical analysis of the macrophage polarization presented in (**A**). Data were analyzed with one-way ANOVA (n = 6 per group). (**D**) Macrophage polarization was assessed in vitro using RAW264.7 cells. Cells were first exposed to *C. albicans* for 1 h, followed by sodium new houttuyfonate treatment for either 2 or 6 h. Changes in polarization were illustrated by representative flow cytometry profiles. Color scale represents cell density (events) (**E**,**F**) Statistical analysis of the macrophage polarization presented in (**D**). Results indicate that sodium new houttuyfonate enhances fungal clearance by temporally regulating the macrophage transition from the anti-inflammatory to the proinflammatory phenotype. Data were analyzed with one-way ANOVA (n = 3 per group). ns: not significant; * *p* < 0.05, ** *p* < 0.01, **** *p* < 0.0001, significantly different from Ca group.

To determine whether sodium new houttuyfonate regulates macrophage functional polarization during antifungal responses, macrophage polarization markers were analyzed following sodium new houttuyfonate stimulation. An *in vivo* flow-cytometric analysis demonstrated that at day 3 post-treatment, the Ca group showed elevated CD86 expression but reduced CD206 expression, indicating a robust proinflammatory state (Figure 4A). However, both high- and low-dose sodium new houttuyfonate treatments significantly upregulated CD206 expression while downregulating CD86 expression, suggesting a transition to an anti-inflammatory state (Figure 4B). By day 7, the Ca group showed high CD206 and low CD86 expression, indicating potential immunosuppression. In contrast, a higher sodium new houttuyfonate dose (20 mg/kg) strongly increased CD86 expression. Treatments with 10 mg/kg sodium new houttuyfonate or FCZ also raised CD86 levels while reducing CD206, reflecting reactivation of the immune system and supporting the clearance of remaining fungal cells. Furthermore, the 20 mg/kg sodium new houttuyfonate group showed a significant increase in double-positive macrophages, corroborating the immunomodulatory effects of sodium new houttuyfonate (Figure 4C).

*In vitro* experiments demonstrated that sodium new houttuyfonate significantly downregulated the expression of CD86, a marker of M1 macrophages, during the early stage of infection (2 h postinfection). By 6 h postinfection, the sodium new houttuyfonate-treated macrophages maintained elevated CD86 levels, whereas CD206 expression was significantly suppressed (Figure 4D–F). These findings indicate that sodium new houttuyfonate promotes macrophage phenotypic switching, facilitating a transition from anti-inflammatory to pro-inflammatory immune responses, thereby enhancing fungal clearance and improving immune function.

### 2.6. Sodium New Houttuyfonate Reprograms Macrophage Functional Homeostasis via Time-Dependent Immunomodulation

**Figure 5:**
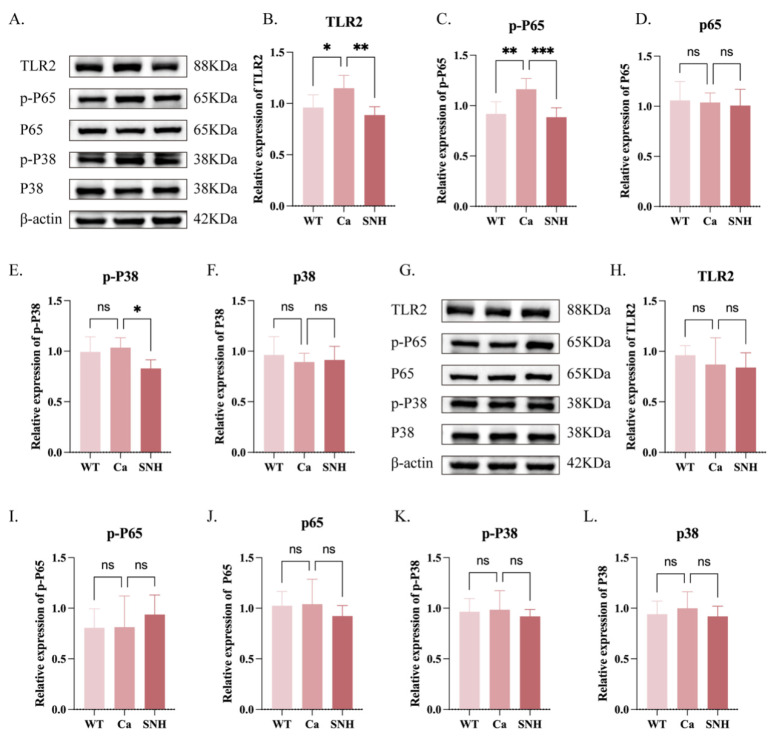
Sodium new houttuyfonate (SNH) reprograms macrophage immune responses through the temporal regulation of signaling pathway activity. (**A**) Expression changes in proteins associated with the TLR2, MAPK, and NF-κB pathways following 2 h of sodium new houttuyfonate treatment. (**B**–**F**) Grayscale value analysis of each protein. (**G**) Expression changes in proteins associated with the TLR, MAPK, and NF-κB pathways following 6 h of sodium new houttuyfonate treatment. (**H**–**L**) Grayscale value analysis of each protein. Data were analyzed with one-way ANOVA. ns: not significant; * *p* < 0.05, ** *p* < 0.01, *** *p* < 0.001, significantly different from Ca group.

To further elucidate the molecular pathways responsible for sodium new houttuyfonate-induced macrophage activation, the involvement of TLR2/NF-κB/MAPK signaling pathways was investigated. In the early stages of infection (2 h post-infection), *C. albicans* infection led to a significant upregulation of TLR2 and p-p65 expression, triggering the immune response and activating the NF-κB signaling pathway. sodium new houttuyfonate treatment effectively downregulated the expression of TLR2, p-p65, and p-p38, suggesting that sodium new houttuyfonate mitigates excessive activation of the TLR2/NF-κB pathway, thereby reducing the inflammatory response and promoting immune homeostasis (Figure 5A–F). In the later stages of infection (6 h post-infection), no significant changes were observed in TLR2, p-p65, or p-p38 expression, indicating that the immune response had entered a steady-state or tolerance phase, with the immune system returning to baseline levels (Figure 5G–L). As a result, the intervention of sodium new houttuyfonate showed limited effects at this stage. These findings demonstrate that sodium new houttuyfonate exerts its protective effects by modulating immune pathways early in infection, preventing excessive inflammation. In contrast, as the immune system adapts or becomes tolerant in the later stages, the therapeutic impact of sodium new houttuyfonate diminishes. Collectively, these results highlight the time-dependent nature of sodium new houttuyfonate’s immune-modulatory effects, with intervention at different stages of infection significantly enhancing the effectiveness of antifungal therapy.

### 2.7. TLR2 Is Essential for Sodium New Houttuyfonate-Mediated Enhancement of Macrophage Antifungal Activity

**Figure 6:**
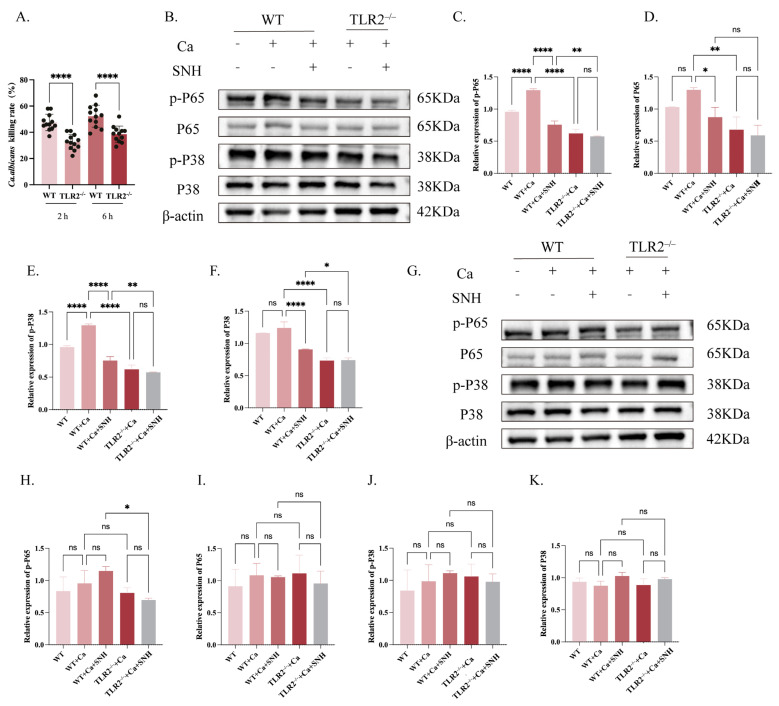
TLR2 is required for sodium new houttuyfonate-mediated enhancement of macrophage antifungal activity and regulation of signaling pathways. (**A**) CFU assay of *C. albicans* survival after co-incubation with WT or TLR2-deficient RAW264.7 macrophages treated with 10 μg/mL sodium new houttuyfonate for 2 h and 6 h. (**B**) Expression of MAPK- and NF-κB-associated proteins at 2 h post-infection. (**C**–**F**) Densitometric analysis of p-p65, p65, p-p38, and p38 at the 2 h time point. (**G**) Expression of MAPK- and NF-κB-associated proteins at 6 h post-infection. (**H**–**K**) Densitometric analysis of p-p65, p65, p-p38, and p38 at the 6 h time point. Data were analyzed with one-way ANOVA. ns: not significant; * *p* < 0.05, ** *p* < 0.01, **** *p* < 0.0001, significantly different between groups as indicated.

To clarify how TLR2 contributes to the immunomodulatory effects of sodium new houttuyfonate, RAW264.7 macrophages lacking TLR2 were prepared and then exposed to *C. albicans*, followed by sodium new houttuyfonate treatment. CFU assays revealed that, compared with wild-type (WT) macrophages, TLR2 deficiency significantly impaired antifungal activity. At both 2 h and 6 h following exposure to 10 μg/mL sodium new houttuyfonate, TLR2-deficient macrophages exhibited reduced fungal killing compared with WT cells, suggesting an important role for TLR2 in sodium new houttuyfonate-mediated clearance of fungi (Figure 6A).

To explore the mechanisms involved, Western blotting was performed to assess the activation status of the NF-κB and MAPK pathways. At the early stage of infection (2 h), *C. albicans* stimulation significantly increased the phosphorylation levels of p65 and p38 in WT macrophages, indicating rapid activation of inflammatory signaling. Sodium new houttuyfonate treatment markedly reduced the expression of p-p65, total p65, p-p38, and total p38 in WT + Ca cells, suggesting that sodium new houttuyfonate effectively suppresses excessive inflammatory responses during early infection.

In contrast, TLR2 deficiency led to a substantial reduction in both basal and infection-induced activation of these signaling molecules. No clear differences were detected between the TLR2^−/−^ + Ca and TLR2^−/−^ + Ca + sodium new houttuyfonate groups, suggesting that the influence of sodium new houttuyfonate on NF-κB/MAPK signaling is largely dependent on the presence of TLR2. Furthermore, compared with WT + Ca + sodium new houttuyfonate cells, TLR2^−/−^ + Ca+ sodium new houttuyfonate macrophages exhibited significantly lower levels of p-p65, p65, p-p38, and p38, further confirming that TLR2 acts as a key upstream mediator of sodium new houttuyfonate-induced signaling modulation (Figure 6B–F).

At the late stage of infection (6 h), sodium new houttuyfonate treatment in WT macrophages showed a trend toward increased phosphorylation of p65 and p38, suggesting reactivation of immune signaling. However, loss of TLR2 markedly reduced this effect, which was accompanied by a significant decrease in p-p65 expression in TLR2^−/−^ + Ca+ sodium new houttuyfonate macrophages compared with WT + Ca + sodium new houttuyfonate cells (Figure 6G–K).

## 3. Discussion

As an opportunistic fungal pathogen, *C. albicans* can disrupt host immune barriers, particularly at mucosal and intestinal sites, and subsequently disseminate systemically, leading to severe complications such as peritonitis, sepsis, multiple organ dysfunction, and even death [1,31,32,33,34]. The rapid progression and high mortality associated with invasive candidiasis highlight the urgent need for effective therapeutic strategies. As key components of innate immunity, Macrophages recognize PAMPs on fungal surfaces, phagocytose invading pathogens, and secrete cytokines that recruit neutrophils and other immune cells to enhance antifungal responses [35,36,37]. In addition, macrophage polarization toward M1 or M2 phenotypes contributes to the regulation of inflammation and coordination of systemic immune defenses [26]. However, impaired macrophage function in immunocompromised hosts may compromise fungal recognition and clearance, thereby facilitating uncontrolled infection [38,39,40]. Moreover, *C. albicans* has evolved multiple immune evasion mechanisms, including macrophage depletion, suppression of glycolysis, and inhibition of NLRP3 inflammasome activation [27,41]. Pattern-recognition receptors, particularly TLR2 and TLR4, are essential for sensing fungal components and activating downstream NF-κB and MAPK signaling pathways, thereby promoting inflammatory responses and antifungal immunity [42,43,44]. Previous studies have demonstrated that activation of TLR signaling enhances macrophage-mediated fungal clearance [45,46,47]. Consistent with these observations, our intraperitoneal *C. albicans* infection model reproduced several pathological features of intra-abdominal candidiasis (Figure 1). Notably, sodium new houttuyfonate treatment significantly increased leukocyte numbers and improved fungal clearance in infected mice. In addition, sodium new houttuyfonate regulated macrophage polarization, promoted inflammatory cytokine secretion and ROS production, and enhanced macrophage-mediated antifungal activity against *C. albicans*. These findings suggest that sodium new houttuyfonate exerts host-directed protective effects by modulating innate immune responses and may represent a promising therapeutic strategy for invasive candidiasis in the face of increasing antifungal resistance.

Sodium new houttuyfonate has unique advantages over conventional antifungal agents (e.g., FCZ), including its natural composition, low toxicity, immunomodulatory properties, and anti-drug resistance [18,22]. In murine models of pathogen-induced infection, sodium new houttuyfonate has demonstrated significant therapeutic efficacy, effectively mitigating tissue damage while enhancing macrophage function through immunomodulation [20,29]. Unlike crude extracts of Houttuynia cordata, sodium new houttuyfonate is a chemically defined single-compound derivative of houttuynin. Previous studies have shown that sodium new houttuyfonate possesses anti-inflammatory and immunomodulatory properties, partly through regulating cytokine production (e.g., IL-1β, IL-6, and TNF-α) and modulating NF-κB and MAPK signaling pathways [9,48]. In previous studies, we demonstrated that prophylactic sodium new houttuyfonate administration significantly enhanced the host’s immune responses, effectively preventing *A. fumigatus* and *C. albicans* infections [20,22,49]. In the present study, sodium new houttuyfonate significantly modulated the TLR2 and TLR4 receptors and their downstream signaling cascades, reversing pathogen-induced immune tolerance and enhancing the macrophage-mediated immune responses.

ROS and inflammatory cytokines play pivotal roles in mediating the phagocytic and killing functions of macrophages during *C. albicans* infection. ROS not only contribute directly to fungal killing but also modulate the immune responses, thereby promoting immune-cell activation and migration [50]. Inflammatory cytokines, such as TNF-α, IL-6, and IL-1β, enhance macrophage phagocytosis, modulate the local immune microenvironment, and thus facilitate pathogen clearance [22,51]. In intra-abdominal *C. albicans* infection, ROS generation correlates closely with the magnitude of the immune responses. In the present study, sodium new houttuyfonate did not simply suppress or enhance inflammation during *C. albicans* infection; rather, it dynamically regulated the balance between antifungal immunity and inflammatory injury in a time-dependent manner. This finding is consistent with previous studies showing that sodium new houttuyfonate or sodium houttuyfonate can modulate macrophage function and restrain excessive inflammatory responses through TLR4/NF-κB-related pathways in bacterial infection or LPS-induced inflammation models [9,29,52]. However, unlike these studies, which mainly emphasized anti-inflammatory effects during bacterial stimulation, our data reveal a biphasic immunomodulatory pattern in fungal infection: sodium new houttuyfonate suppressed excessive TLR2/NF-κB/MAPK activation and reduced several inflammatory mediators during the early phase, while enhancing macrophage antifungal activity, ROS production, iNOS expression, and pro-inflammatory cytokine release during the later phase.

Our findings also extend previous reports on the antifungal activity of sodium new houttuyfonate against *C. albicans*. Wu et al. reported that sodium new houttuyfonate inhibited *C. albicans* biofilm formation by suppressing the Ras1-cAMP-Efg1 pathway, suggesting a direct effect on fungal morphogenesis and biofilm development [53]. In contrast, our XTT and macrophage-killing assays indicate that, under the present experimental conditions, sodium new houttuyfonate-mediated protection was mainly associated with host-directed immune regulation rather than direct fungicidal activity. Thus, together with earlier work, our results suggest that sodium new houttuyfonate may exert antifungal effects through both pathogen-directed and host-directed mechanisms, depending on the infection model, fungal growth state, and experimental context.

The late-stage macrophage response observed at 6 h should also be interpreted in light of previous studies on *C. albicans*–macrophage interactions. *C. albicans* can undergo hyphal transition after phagocytosis, promote macrophage injury, and escape from phagocytes during prolonged co-incubation [54,55]. Therefore, the 6 h time point likely reflects a more complex host–pathogen interaction than simple pathogen recognition. Nevertheless, the coordinated changes observed across macrophage polarization, cytokine secretion, ROS generation, and fungal killing support the biological relevance of this later response. Although sodium new houttuyfonate no longer markedly altered TLR2 expression or downstream NF-κB/MAPK phosphorylation at 6 h, this does not indicate that TLR2 was dispensable. Instead, TLR2 may act as a permissive upstream receptor that maintains macrophage responsiveness to sodium new houttuyfonate and supports downstream antifungal effector programs.

Importantly, the role of TLR2 in candidiasis remains controversial and context-dependent. Netea et al. reported that *C. albicans* can exploit TLR2-derived signals to promote IL-10 production and regulatory T-cell survival, thereby suppressing antifungal immunity [56]. In contrast, our data showed that sodium new houttuyfonate-treated macrophages exhibited enhanced ROS generation, increased iNOS expression, elevated antifungal activity, and improved fungal clearance despite the induction of IL-10. This suggests that, in our model, TLR2 signaling predominantly contributed to protective macrophage activation rather than tolerogenic immune suppression. The concomitant increase in IL-10 may represent a compensatory regulatory mechanism that limits excessive tissue injury while preserving antifungal defense. Therefore, our findings refine previous observations by suggesting that sodium new houttuyfonate may reshape TLR2-associated responses toward a more balanced and protective immune state during *C. albicans* infection.

In addition, it is also possible that sodium new houttuyfonate-mediated immunomodulation involves receptor crosstalk rather than a strictly linear TLR2-dependent pathway. Previous studies have shown that sodium new houttuyfonate can regulate host immune responses through the TLR4/NF-κB pathway during *P. aeruginosa* infection, while other reports suggest that sodium new houttuyfonate may affect *C. albicans* through the Ras1-cAMP signaling pathway [9,29,53]. In addition, our preliminary observations indicate that TLR2 deficiency is associated with increased expression of the scavenger receptors MSR1, MARCO, and CD36, suggesting the existence of compensatory receptor mechanisms that may participate in sodium new houttuyfonate recognition or downstream immune regulation. Therefore, TLR2 should be regarded as an important, but not necessarily exclusive, mediator of sodium new houttuyfonate-induced macrophage reprogramming. Several limitations should also be acknowledged. Although our data suggest that sodium new houttuyfonate exerts time-dependent immunomodulatory effects during *C. albicans* infection, the precise cellular events occurring during prolonged macrophage–fungus interactions remain incompletely understood. Furthermore, the present study cannot fully distinguish the relative contributions of fungal adaptation, receptor crosstalk, and host immune regulation to the responses observed at 6 h (Figure 4). Future studies will be valuable for further validating and refining the temporal immunoregulatory model proposed here.

From a translational perspective, the doses used in the present study (10 and 20 mg/kg in mice) correspond to human equivalent doses (HEDs) of approximately 0.81 and 1.62 mg/kg, respectively, based on the FDA-recommended body surface area conversion method and the approach proposed by Reagan-Shaw et al. [57]. For a 60 kg adult, these doses correspond to approximately 49–97 mg/day. These estimates suggest that the experimental dosing regimen falls within a potentially achievable clinical range. However, the pharmacokinetic characteristics, bioavailability, tissue distribution, and safety profile of sodium new houttuyfonate in humans remain incompletely characterized. Therefore, although the present findings support the potential translational value of sodium new houttuyfonate as an immunomodulatory agent against *Candida* infection, dedicated pharmacokinetic, toxicological, and dose-escalation studies will be required to further evaluate its clinical feasibility and therapeutic applicability.

## 4. Materials and Methods

### 4.1. Materials and Reagents

Dulbecco’s modified Eagle’s medium (DMEM) and fetal bovine serum were obtained from Gibco (Grand Island, NY, USA). Antibodies directed against β-actin, TLR2, p38 beta/MAPK11 + p38 alpha/MAPK14, phosphorylated p38 (phospho T180 + Y182), NF-κB p65 (phospho S536), NF-κB p65, and iNOS were provided by Abcam (Wales, UK). Cell-staining reagents, including fixable viability stain 780, Fc block, labeled antibodies, and the BD Cytofix/Cytoperm™ Fixation/Permeabilization Kit, were provided by BD Biosciences (Franklin Lakes, NJ, USA). CCK-8 and DCFH-DA were provided by APExBIO (Houston, TX, USA). The Enhanced BCA Protein Assay Kit (P0009) was provided by Beyotime Biotechnology (Shanghai, China). Sodium new houttuyfonate (purity > 98%) was supplied by Fengyao Tonghui Chemical Company (Wuhan, China) and used as a chemically defined single compound throughout the study. Sodium new houttuyfonate stock solutions were prepared in sterile water containing 0.05% Tween-80 to improve solubility and were diluted with the corresponding culture medium immediately before use. The stock solution was diluted to the desired working concentrations immediately before use. Vehicle control groups received the same final concentration of Tween-80 without sodium new houttuyfonate. Fluconazole (FCZ) was from Macklin Chemical Company (Shanghai, China).

### 4.2. Fungal Strains and Culture

In this study, we used the standard *C. albicans* strain ATCC MYA-2876, maintained at the Laboratory of Pathogenic Fungal Infection and Immune Response, Southwest Medical University, Luzhou, Sichuan, China. The strain was cultured in yeast extract peptone dextrose (YPD) medium for amplification. For each experiment, a single preactivated colony was picked from a YPD agar plate and transferred into liquid YPD medium, then cultured at 37 °C for 16–18 h to fully activate the cells.

### 4.3. Establishment of a Murine Model

In this study, we used male wild-type BALB/c mice obtained from Chongqing Tengxin Biotechnology Co., Ltd. (Chongqing, China). All animal experiments were conducted with approval from the Experimental Animal Ethics Committee of Southwest Medical University (approval no.: 20220817-016, 17 August 2022). The mouse model of intra-abdominal candidiasis and the sodium new houttuyfonate dosing protocols were established based on our team’s previous publications [22]. In the therapeutic model of peritoneal *Candida* infection, mice were randomized into five groups (n = 6 per group): (1) the control group, which received an intraperitoneal injection of 100 μL sterile DMEM containing 0.05% Tween-80, followed by an equal volume of vehicle solution 2 h later; (2) the *C. albicans* (Ca) infection group, which received an intraperitoneal injection of 100 μL of *C. albicans* suspension (2 × 10^8^ cells/mL), followed 2 h later by 100 μL sterile DMEM containing 0.05% Tween-80 (vehicle control); (3) the low-dose sodium new houttuyfonate group (10 mg/kg), which received *C. albicans* as described for Group 2, followed 2 h later by 100 μL sodium new houttuyfonate (10 mg/kg); (4) the high-dose sodium new houttuyfonate group (20 mg/kg), which received *C. albicans* as described for Group 2, followed 2 h later by 100 μL sodium new houttuyfonate (20 mg/kg); and (5) the FCZ group, which received *C. albicans* as described above, followed 2 h later by 100 μL fluconazole (12 mg/kg). The mice were euthanized on day 3 or 7 postinfection for tissue collection and subsequent analysis.

Blood samples were collected with orbital bleeding into EDTA-anticoagulated tubes for comprehensive blood cell analysis. Liver, kidney, small-intestinal, and large-intestinal tissues were split into two equal portions: one was subjected to fungal load measurement, whereas the other was fixed in 4% paraformaldehyde and processed for paraffin embedding. For histological evaluation, liver samples were stained with hematoxylin and eosin (H&E), whereas kidney, small-intestinal, and large-intestinal tissues were subjected to PAS staining.

The fungal burden was normalized to tissue weight and expressed as colony-forming units per gram of tissue (CFU/g). The fungal load was calculated according to the following formula:

CFU/g tissue = (Number of colonies × dilution factor × homogenate volume)/tissue weight (g)

where the number of colonies represents the colony count obtained from YPD agar plates, the dilution factor corresponds to the serial dilution used for plating, and tissue weight refers to the wet weight of the harvested organ sample.

### 4.4. Peritoneal Macrophage Isolation and Polarization Analysis

Murine peritoneal macrophages were isolated with established protocols [58]. Mice were deeply anesthetized with sodium pentobarbital prior to terminal retro-orbital blood collection. Following orbital blood collection, euthanasia was performed with cervical dislocation. The mice were then positioned supine on a dissection board with their limbs secured and injected intraperitoneally with 5–10 mL of prechilled phosphate-buffered saline (PBS). The abdominal surface was disinfected with 75% ethanol for 1–2 min to minimize contamination during the procedure. The experiments were optimized based on a previously published study [59].

### 4.5. Fungal Proliferation Inhibition Assay

The procedure was conducted using a protocol reported in our earlier work [22]. *C. albicans* suspensions were treated with serial dilutions of sodium new houttuyfonate (2.5–320 μg/mL). The suspensions were brought to 1 × 10^6^ Cells/mL and subsequently seeded into 96-well culture plates at 100 μL per well. The plates were incubated statically for 6 h at 37 °C under aerobic conditions. Following incubation, OD_600_ was determined using a microplate reader. The fungal proliferation inhibition rates were calculated relative to the untreated control group. The experiment was independently replicated three times, and the data are presented as means ± standard deviations (SD).

### 4.6. Cell Proliferation Assay

Cell proliferation was evaluated using a CCK-8 assay following the kit instructions. RAW264.7 cells were plated in 96-well plates at a density of 1 × 10^4^ cells per well in 100 μL medium. After attachment and stabilization, the culture medium was gently aspirated and replaced with fresh medium containing the indicated concentrations of sodium new houttuyfonate. Following treatment, the culture medium was removed, and CCK-8 working solution was added directly according to the manufacturer’s instructions. Subsequently, cells were treated with 100 μL of 10% CCK-8 solution and incubated at 37 °C for 1 h, after which absorbance was recorded at 450 nm. All medium changes were performed by gentle aspiration and replacement to minimize disturbance of the adherent RAW264.7 monolayer.

### 4.7. Flow-Cytometric Analysis of Cell-Surface Markers

For the *in vivo* analysis of peritoneal macrophages, peritoneal lavage cells were collected from mice, washed with PBS, and resuspended in staining buffer. Cells were first stained with BD Pharmingen™ Fixable Viability Stain 780 (Becton, Dickinson and Company, Franklin Lakes, NJ, USA) to exclude dead cells, followed by Fc receptor blocking according to the manufacturer’s instructions. Surface staining was then performed using anti-CD45, anti-CD11b, anti-F4/80, and anti-CD86 antibodies for 30 min at 4 °C in the dark. After washing, cells were fixed and permeabilized using the BD Cytofix/Cytoperm™ Fixation/Permeabilization Kit (BD Biosciences) according to the manufacturer’s protocol. Briefly, cells were incubated with 250 μL of fixation/permeabilization solution for 20 min at 4 °C, washed with 1× Perm/Wash buffer, and subsequently stained with anti-CD206 antibody for 30 min at 4 °C in the dark. Peritoneal macrophages were identified as live CD45^+^CD11b^+^F4/80^+^ cells, and macrophage polarization was evaluated based on CD86 and CD206 expression.

For the *in vitro* analysis, RAW264.7 cells were harvested after the indicated treatments, washed with PBS, and stained with Fixable Viability Stain 780. Because RAW264.7 cells are an established macrophage cell line, CD45, CD11b, and F4/80 gating strategies were not applied. Cells were stained directly with anti-CD86 antibody for 30 min at 4 °C in the dark, followed by fixation and permeabilization using the BD Cytofix/Cytoperm™ Fixation/Permeabilization Kit. After washing with Perm/Wash buffer, intracellular CD206 staining was performed for 30 min at 4 °C in the dark. Finally, cells were resuspended in staining buffer and analyzed using a BD flow cytometer (Becton, Dickinson and Company, Franklin Lakes, NJ, USA). Data were processed using FlowJo software (version 10.8.1; Tree Star, Inc., Ashland, OR, USA).

### 4.8. Real-Time Quantitative PCR (qPCR) Analysis

To evaluate the sodium houttuyfonate-induced transcriptional changes, RAW264.7 cells cultured in vitro were harvested and subjected to qPCR analysis according to a previously described protocol along with the manufacturer’s instructions [20]. Total RNA was extracted with RNAiso™ reagent (Takara, Dalian, China), and the concentration was measured with a NanoDrop™ One Spectrophotometer (Thermo Fisher Scientific, Shanghai, China). cDNA synthesis and subsequent qPCR were performed with reagents (Takara, Dalian, China), according to the manufacturer’s protocols. Changes in the expression of the genes encoding interleukin 1β (*IL-1β*), interleukin 6 (*IL-6*), tumor necrosis factor α (*TNF-α*), C-C motif chemokine ligand 2 (*CCL2/MCP-1*), *TLR2*, *TLR4*, nuclear factor-kappa B (*NF-κB/P65*), *p38*, mitogen-activated protein kinase 1 (ERK/Mapk1), and mitogen-activated protein kinase 8 (JNK/Mapk8) were analyzed. Primer sequences are listed in Supplementary Table S1, and relative expression levels were determined using the 2^−ΔΔCT^ method, using glyceraldehyde 3-phosphate dehydrogenase (GAPDH) transcript levels as the endogenous control. Three technical replicates were included per sample.

### 4.9. Macrophage Phagocytosis and Killing Assay

RAW264.7 murine macrophages were plated in 12-well plates at a density of 2.5 × 10^5^ cells/mL (1 mL per well) and maintained for 24 h at 37 °C in a 5% CO_2_ atmosphere. For phagocytosis and fungal burden assessment, *C. albicans* yeast cells were introduced to RAW264.7 macrophages at a multiplicity of infection (XTT) of 4 and incubated for 1 h to allow uptake. Following incubation, wells were gently rinsed three times with ice-cold PBS (5 min per wash) to remove extracellular, non-internalized yeast cells. Subsequently, three wells were randomly selected, and cells were lysed with 1 mL sterile ultrapure water at 4 °C for 15 min. The lysates were collected and centrifuged at 500× *g* for 5 min, after which the supernatants were discarded and the pellets were resuspended and diluted stepwise in PBS. Aliquots (100 μL) of each dilution were plated onto YPD agar plates and incubated at 37 °C for 24 h. CFU were then enumerated and recorded as N_0_. The remaining wells contained DMEM (negative control) or sodium new houttuyfonate (2.5, 5, or 10 μg/mL). After incubation for 1, 2, or 6 h, the cells were lysed and the viable intracellular fungi quantified (N_t_).

The macrophage killing efficiency was calculated as: killing efficiency = (N_0_ − N_t_)/N_0_ × 100%. CFU concentration (CFU/mL) = counted CFU × (total volume/plated volume)× dilution factor.

### 4.10. DCFH-DA-Based Detection of Cellular ROS

Experimental procedures were carried out with minor modifications based on the instructions provided with the Reactive Oxygen Species Assay Kit (Beyotime Biotechnology, Shanghai, China). RAW264.7 macrophages were prepared at a density of 2.5 × 10^5^ cells/mL and seeded into 12-well plates containing sterilized glass coverslips (1 mL per well) or into 96-well plates (100 μL per well).

The cells were precultured for 24 h at 37 °C under 5% CO_2_ and 95% humidity until they reached 80% confluence. Activated *C. albicans* yeast cells suspended in serum-free DMEM at a concentration of 1 × 10^6^ Cells/mL were added at an effector-to-target ratio of 1:4, followed by incubation at 37 °C with gentle rotation at 30 rpm. After coincubation for 1 h, any extracellular yeast cells were removed with three consecutive washes with ice-cold PBS, each performed for 5 min at 100 rpm.

The cells were divided into four experimental groups: Group A, treated with DMEM (negative control); and Groups B to D, treated with sodium new houttuyfonate dissolved in DMEM at concentrations of 2.5, 5, and 10 μg/mL, respectively. The treatment volumes were standardized according to the plate format, with 500 μL/well added to the 12-well plates and 100 μL/well to the 96-well plates. After incubation for precisely 1 or 2 h (allowable variation ± 5 m), the culture media were aspirated. The cells were then incubated with DCFH-DA working solution prepared at 10 μmol/L in serum-free DMEM and prewarmed to 37 °C, using loading volumes of 1 mL/well for the 12-well plates and 100 μL/well for the 96-well plates. After incubation at 37 °C for 25 min in the dark, excess DCFH-DA was removed by washing the adherent cells three times with prewarmed serum-free DMEM. Washing was performed by gentle aspiration of the medium followed by careful addition of fresh medium along the wall of each well to minimize disturbance of the adherent macrophage monolayer. For the 12-well-plate samples, fluorescence was visualized directly with fluorescence microscopy (ex = 488 nm, em = 525 nm). ROS-positive cells were quantified in five random fields per coverslip. For the 96-well-plate samples, the real-time fluorescence intensity was monitored with a microplate reader at identical wavelengths. The final fluorescence intensity was calculated as: net FI = FI_sample_ − FI_blank_.

### 4.11. Western Blotting Analysis

This experiment was performed according to a published experimental method [22]. Total protein was extracted from tissue samples using ice-cold RIPA lysis buffer (containing 1× protease and phosphatase inhibitor cocktail) obtained from Yamei Biological Pharmaceutical Technology Co., Ltd. (Shanghai, China). Protein levels were quantified using a BCA-based assay. For electrophoresis, equal amounts of protein (30 μg per lane) were loaded onto 10% SDS–polyacrylamide gels (1.0 mm) and resolved before being transferred to PVDF membranes (0.45 μm) under 100 V for 60 min at 4 °C. After protein transfer, membranes were blocked according to the target proteins. Membranes used for the detection of phosphorylated proteins (including phospho-p38 and phospho-p65) were blocked with 5% bovine serum albumin (BSA) in TBS-T for 2 h at room temperature, whereas membranes used for total proteins and β-actin were blocked with 5% skim milk in TBS-T under the same conditions. Subsequently, membranes were incubated overnight at 4 °C with the corresponding primary antibodies diluted 1:1000 in 5% BSA/TBS-T. Membranes were rinsed three times for 10 min each in TBS-T with gentle agitation (60 rpm), the membranes were incubated with horseradish peroxidase (HRP)-conjugated secondary antibody (goat anti-rabbit IgG or goat anti-mouse IgG) diluted 1:2000 in blocking buffer for 2 h at room temperature. After five 10 min washes with TBS-T, the protein bands were visualized with Super Signal™ West Pico PLUS Chemiluminescent Substrate (Thermo Fisher Scientific, Shanghai, China), with exposure times ranging from 10 s to 10 min. Signal intensities were analyzed with ImageJ (v1.53) and expressed relative to β-actin.

### 4.12. Statistical Analysis

Statistical analyses were performed using GraphPad Prism 8.0. All data are presented as the mean ± SD from at least three independent experiments. For comparisons among multiple treatment groups at the same time point, one-way ANOVA followed by Tukey’s multiple comparisons test was used. The 2 h and 6 h datasets were analyzed separately because comparisons were made only among different treatments within each time point. A *p*-value < 0.05 was considered statistically significant. Significance levels are indicated in the figures as follows: * *p* < 0.05; ** *p* < 0.01; *** *p* < 0.001; and **** *p* < 0.0001.

## 5. Conclusions

**Figure 7:**
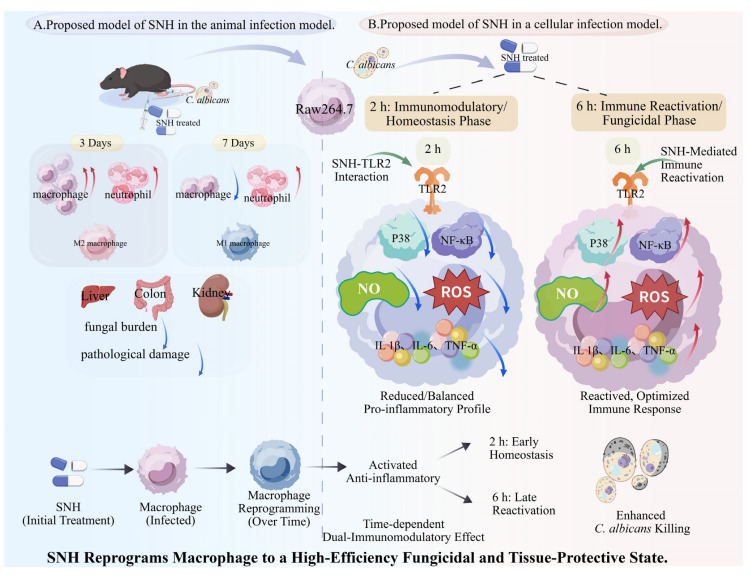
Schematic diagrams of the proposed models of sodium new houttuyfonate treatment for *C. albicans* infection. (**A**) Schematic representation of sodium new houttuyfonate treatment in an in vivo infection model. A murine intraperitoneal candidiasis model was established, and sodium new houttuyfonate administration was initiated 2 h post-infection and continued for 3 or 7 days. The polarization of peritoneal macrophages, the fungal loads in various tissues, and histopathological damage were analyzed. (**B**) A schematic model illustrating the role of sodium new houttuyfonate in a cellular infection system. RAW264.7 cells were exposed to *C. albicans* for 1 h, followed by treatment with sodium new houttuyfonate for 2 or 6 h. The assayed parameters included macrophage polarization, fungal phagocytosis and killing, ROS and NO production, cytokine secretion, and the activation of relevant signaling pathways. Red upward arrows indicate upregulation; blue downward arrows indicate downregulation.

Overall, sodium new houttuyfonate was found to reduce fungal load and ease tissue injury in a mouse model of intra-abdominal *C. albicans* infection. Its antifungal efficacy is primarily mediated through host-directed immunomodulation rather than direct fungicidal activity. Mechanistically, sodium new houttuyfonate regulates macrophage function via TLR2-dependent NF-κB/MAPK signaling, suppressing excessive inflammation during the early stage of infection while promoting immune reactivation at later stages to enhance pathogen clearance. Notably, TLR2 deficiency markedly attenuated the immunomodulatory and antifungal effects of sodium new houttuyfonate, confirming its essential role in this process. Collectively, our data suggest that sodium new houttuyfonate may function as a host-targeted antifungal agent and highlight the possible value of Chinese patent medicines for treating drug-resistant fungal infections (Figure 7).

## Data Availability

All data generated or analyzed during this study are included in this article. Further enquiries can be directed to the corresponding author.

## Supplementary Materials

The following supporting information can be downloaded at: https://www.mdpi.com/article/10.3390/ijms27146437/s1.

## Abbreviations

µ	micro
°C	degrees Celsius
*C. albicans*	*Candida albicans*
CCK8	Cell Counting Kit-8
cDNA	complementary deoxyribonucleic acid
CFU	Colony-forming units
DCFH-DA	2,7-Dichlorodi -hydrofluorescein diacetate
DMSO	Dimethyl sulfoxide
DNA	deoxyribonucleic acid
FCZ	Fluconazole
IAC	Intra-abdominal candidiasis
iNOS	Inducible nitric oxide synthase
MAPK	Mitogen-activated protein kinase
min	minute(s)
mL	milliliter
mRNA	messenger RNA
NF-κB	Nuclear factor kappa-B
NO	Nitric oxide
NS	Not significant
PAMPs	Pathogen-associated molecular patterns
PAS	Peripheral anionic site
PBS	Phosphate buffer saline
PRRs	Pattern recognition receptors
qRT-PCR	Quantitative real-time PCR
ROS	Reactive oxygen species
rpm	revolutions per minute
SNH	Sodium new houttuyfonate
TLR	toll-like receptor
TNF-α	tumor necrosis factor-alpha
WB	Western blot
WT	wild type
YPD	Yeast extract peptone dextrose medium
