# Supplementary material for: Therapeutic Effects and Mechanisms of Sodium New Houttuyfonate in a Murine Model of Intra-Abdominal Candida albicans Infection
Source: Int J Mol Sci. 2026 Jul 20;27(14):6437. doi: 10.3390/ijms27146437 (PMC13410132; doi:10.3390/ijms27146437)
Supplement: Supplementary file 1 [file ijms-27-06437-s001.zip › ijms-4326516-supplementary.pdf]

Table S1 Primer sequence for real-time quantitative PCR

Primer sequences of RT-qPCR

Primer Name	Primer Sequence (5'-3')
GAPDH-F	AATGTGTCCGTCGTGGATCT
GAPDH-R	AGACAACCTGGTCCTCAGTG
TNF-F	GGGCCTCAAAGGAAAGAATCT
TNF-R	GAGGTGCTGATGTACCAGTTGG
IL 1β-F	TCTGGGGAGGCACATCTTCT
IL 1β-R	CAGGTCCAAGTTGCCGTTTC
IL 10-F	GGCCCAGAAATCAAGGAGCA
IL 10-R	GCCTTGTAGACACCTTGGTCTT
IL 6-F	TGCTTCCCCATCTCTCATGC
IL 6-R	CTGTCTGGAAAAAGTGCCGC
MCP-1-F	TCGCCGCTTAGTCACATACC
MCP-1-R	GGTCACCAGGTACACGTCAT
